# A clinical model to predict successful renal replacement therapy (RRT) discontinuation in patients with Acute Kidney Injury (AKI)

**DOI:** 10.1016/j.clinsp.2023.100280

**Published:** 2023-09-09

**Authors:** Eduardo de Oliveira Valle, Igor Smolentzov, João Lucas Martins Gorzoni, Isabela Cavalcante Salgado, Lorena Catelan Mainardes, Vanessa Oliveira Gomes, Charles Hamilton Mélo Júnior, Camila Eleuterio Rodrigues, José Mauro Vieira Júnior

**Affiliations:** a Nephrology Department, Hospital das Clínicas, Faculdade de Medicina da Universidade de São Paulo, São Paulo, SP, Brazil; b Nephrology Department, Prince of Wales Clinical School ‒ UNSW Medicine & Health, Sydney, Australia

**Keywords:** Hemodialysis, Acute kidney injury, Score, Discontinuation, Catheter removal

## Abstract

**Introduction:**

Ideal timing of Renal Replacement Therapy (RRT) discontinuation in Acute Kidney Injury (AKI) is still unknown. We aimed to study the role of creatinine-related variables in predicting RRT successful discontinuation and to propose a clinical predictive score.

**Methods:**

In this single-centre retrospective study, we evaluated all AKI patients in whom RRT was interrupted for at least 48 hours. Patients who were still RRT-independent 7 days after initial RRT cessation were included in the “Success” group and opposed to the “Failure” group. We evaluated baseline characteristics and variables collected at the time of RRT interruption, as well as the Kinetic estimated Glomerular Filtration Rate (KeGFR), the simple variation in serum Creatinine (ΔsCr), and the incremental creatinine ratio on the first three days after RRT interruption. Multivariable analysis was performed to evaluate prediction of success. Internal validation using a simple binomial generalized regression model with Lasso estimation and 5-fold cross validation method was performed.

**Results:**

We included 124 patients, 49 in the “Failure” group and 75 in the “Success” group. All creatinine-related variables predicted success in simple and multiple logistic regression models. The best model generated a clinical score based on the odds ratio obtained for each variable and included urine output, non-renal SOFA score, fluid balance, serum urea, serum potassium, blood pH, and the variation in sCr values after RRT discontinuation. The score presented an area under the ROC of 0.86 (95% CI 0.76‒1.00).

**Conclusion:**

Creatinine variation between the first 2 consecutive days after RRT discontinuation might predict success in RRT discontinuation. The developed clinical score based on these variables might be a useful clinical decision tool to guide hemodialysis catheter safe removal.

## Highlights

• Score to predict successful RRT discontinuation in AKI.
• Timely catheter removal.
• Comparison among different strategies to assess.
• Creatinine variation.

## Introduction

Renal Replacement Therapy (RRT) is a major supportive treatment offered to patients with severe Acute Kidney Injury (AKI) in Intensive Care Units (ICU). The ideal time to start RRT in patients with AKI has been widely discussed and most clinicians agree that RRT should be commenced when life-threatening changes in electrolyte, acid-base or fluid balance exist.^1^ In the absence of such dangerous disorders, clinical context should be considered.

Despite the fact that RRT provides beneficial support to patient recovery, unnecessary RRT may be harmful. Inappropriate removal of antibiotics and amino acids may occur^2^^,^^3^ and intensive RRT schedules have been associated with delayed kidney recovery and high rates of catheter-related bloodstream infections.^4^^,^^5^

Once RRT has been initiated, the appropriate timing to interrupt RRT and remove the vascular access is still unknown. Previous studies have shown utility in assessing urine output at the time of RRT discontinuation as an important predictor of successful cessation, especially in patients not receiving diuretics.^6^, ^7^, ^8^ Low tubular damage assessed by low values of Urinary Neutrophil Gelatinase-Associated Lipocalin (uNGAL)[9] and better kidney function (assessed by increased urinary creatinine or urea excretion and by creatinine clearance) were also demonstrated to successfully predict RRT discontinuation.^10^, ^11^, ^12^, ^13^ However, urine collection necessary to calculate those parameters may be cumbersome and reduces its clinical utility. The incremental creatinine ratio evaluates the variation in serum creatinine between subsequent days and has sometimes been used as a surrogate of kidney function to avoid inconvenient urine collection necessary to calculate creatinine clearance.^13^ However, despite indicating the direction of kidney function change, the incremental creatinine ratio does not provide the magnitude of Glomerular Filtration Rate (GFR).

Equations using only plasma functional markers, such as creatinine or cystatin C, do not accurately reflect real GFR in non-steady states, such as AKI. To counter these circumstances and determine kidney function in the acute setting, a formula considering acute changes in GFR was developed: the Kinetic Estimated Glomerular Filtration Rate (KeGFR).^14^ High KeGFR values have already been suggested as good predictors for RRT discontinuation, especially when combined with urine output.^15^

The purpose of this study is to find the value of KeGFR and to compare it with the simple variation in serum creatinine between two consecutive days at the time of RRT interruption and with the incremental creatinine ratio (creatinine day 2/day 1 and creatinine day 3/day 1) to predict successful RRT weaning. We aim to assess how all variables interact with other clinical important measures (e.g., fluid status and electrolytes levels) in prediction of successful RRT discontinuation.

## Methods

### Study design and population

This was a single-centre, retrospective, observational study, assessing critically ill patients from Hospital das Clínicas, a large tertiary care hospital in São Paulo, Brazil. All patients receiving RRT due to AKI from October 2020 to February 2022 were considered for inclusion in the study. Modalities of RRT included intermittent haemodialysis, prolonged intermittent RRT and continuous renal replacement therapy.

The criteria for initiation and interruption of RRT were at the discretion of the attending physician. Patients who managed to remain at least 48 consecutive hours without receiving RRT prescription were included. Those who had RRT interrupted exclusively due to hemodynamic instability, death or decision for palliative care were excluded.

Successful RRT discontinuation criteria were met when included patients were alive and free from RRT 7 days after interrupting RRT.^10^, ^11^, ^12^^,^^15^ Patients were then divided in two groups, according to success in RRT discontinuation: Success and Failure.

### Data collection

At ICU admission, clinical and demographic data were retrieved. Baseline serum Creatinine (sCr) was defined as the lowest value from 3 months before admission to one month after discharge and baseline estimated glomerular filtration rate was calculated using CKD-EPI 2021 formula.^16^

Clinical variables that are usually considered as important by clinicians when deciding RRT discontinuation were collected by the time of RRT interruption: levels of serum urea and potassium, levels of blood bicarbonate and pH, Urine Output (UO), daily fluid balance, need of diuretic and overall clinical status determined by the “non-renal” Sequential Organ Failure Assessment (SOFA) score. The “non-renal” SOFA (nrSOFA) was determined by the sum of all components of SOFA score apart from sCr (maximum score value 20). In addition, we calculated nrSOFA variation between the day of RRT initiation and the day of RRT discontinuation (ΔnrSOFA), as a surrogate of improving (if positive) or deteriorating (if negative) overall clinical status of each patient during RRT interruption.

Serum creatinine measurements on the first three consecutive days after RRT discontinuation were used to calculate KeGFR and to calculate the simple variation in serum creatinine (ΔsCr), calculated as the difference in sCr levels between 2 consecutive days after RRT discontinuation adjusted for 24h (e.g., sCr at 2 days after RRT discontinuation – sCr at 1 days after RRT discontinuation/timeframe between those collections, in hours × 24h). We have also calculated the creatinine ratios (day 2/day 1 and day 3/day 1), as suggested by other authors.^13^ The KeGFR formula is derived from the initial amount of creatinine and its production rate, the volume of distribution and the difference in levels of two consecutive plasma creatinine measures over any time.^14^

### Outcomes

The primary outcome is the success status within 7 days after RRT discontinuation, as described above.

### Ethical aspects

This study was approved by CAPPesq, the local institutional human research ethics committee (Reference n° 51678521.0.0000.0068) and was registered in ClinicalTrials.gov under the number NCT06005896. This study was performed following the Strengthening the Reporting of Observational Studies in Epidemiology statement.^17^

### Statistical analysis

Normally distributed continuous variables are reported as mean ± Standard Deviation (SD), skewed-distributed continuous variables as median and Interquartile Range (IQR), and categorical variables are summarised as absolute numbers and proportions. “Success” and “Failure” patients were compared by the appropriate test according to the distribution of continuous variables (analysis of variance or Kruskal-Wallis test), and categorical variables were compared by Fisher's exact test. Differences in means/median are reported and were considered statistically significant if *p* < 0.05.

We used simple and multiple logistic regression models to predict success in RRT discontinuation. In simple logistic regression models, we tested each of all collected variables as predictors and identified those associated with the outcome according to *p*-value significance. Univariable models allowed the generation of Receiver Operating Characteristic (ROC) curves and the assessment of the optimal cut-off values considering the maximum [sensitivity + specificity − 1] of each variable.

Clinically important variables which were also considered as predictors in the univariable models were included in the multivariable models. Multiple binomial logistic regression models were initially performed using continuous predictors. We compared models using KeGFR, ΔsCr and incremental creatinine ratio both from RRT discontinuation up to the following day (D1, or day 2/day 1 ratio) and from one day after RRT discontinuation up to the following day (D2, or day 3/day 1 ratio) using ROC curves. The decision between the best model was made based on the area under the ROC curve.

If no difference existed in model performance according to the ROC curve, we preferred to choose models comprising variables collected at D1 instead of D2, because this would permit more timely clinical decisions and possible earlier catheter removal.

We subsequently created multiple logistic regression models using the same variables included in the model chosen, but categorizing them according to the optimal cutoff values generated at each individual univariable model. We have chosen this approach to simplify interpretation and allow a score generation to guide clinical decisions. The Odds Ratio (OR) obtained for each variable was rounded to the nearest integer to determine the value of each variable at the final discontinuation score.

We applied the final discontinuation score to each patient in this cohort, and generated training and internal validation ROC curves to predict success in RRT discontinuation based on a simple binomial generalized regression model with Lasso estimation and 5-fold cross validation method (using only the created score as a predictor). The likelihood of success depending on the final discontinuation score was assessed. We constructed 5000-times bootstrap 95% Confidence Intervals for the areas under the ROC curve regarding training and internal validation. Analyses were performed with the R statistical software, version 4.0.5; Rstudio, version 1.4.1106 (R Development Core Team, 2020) and JMP Pro version 16 (SAS Institute, Cary, NC, USA).

## Results

### Patients

**Fig. 1:**
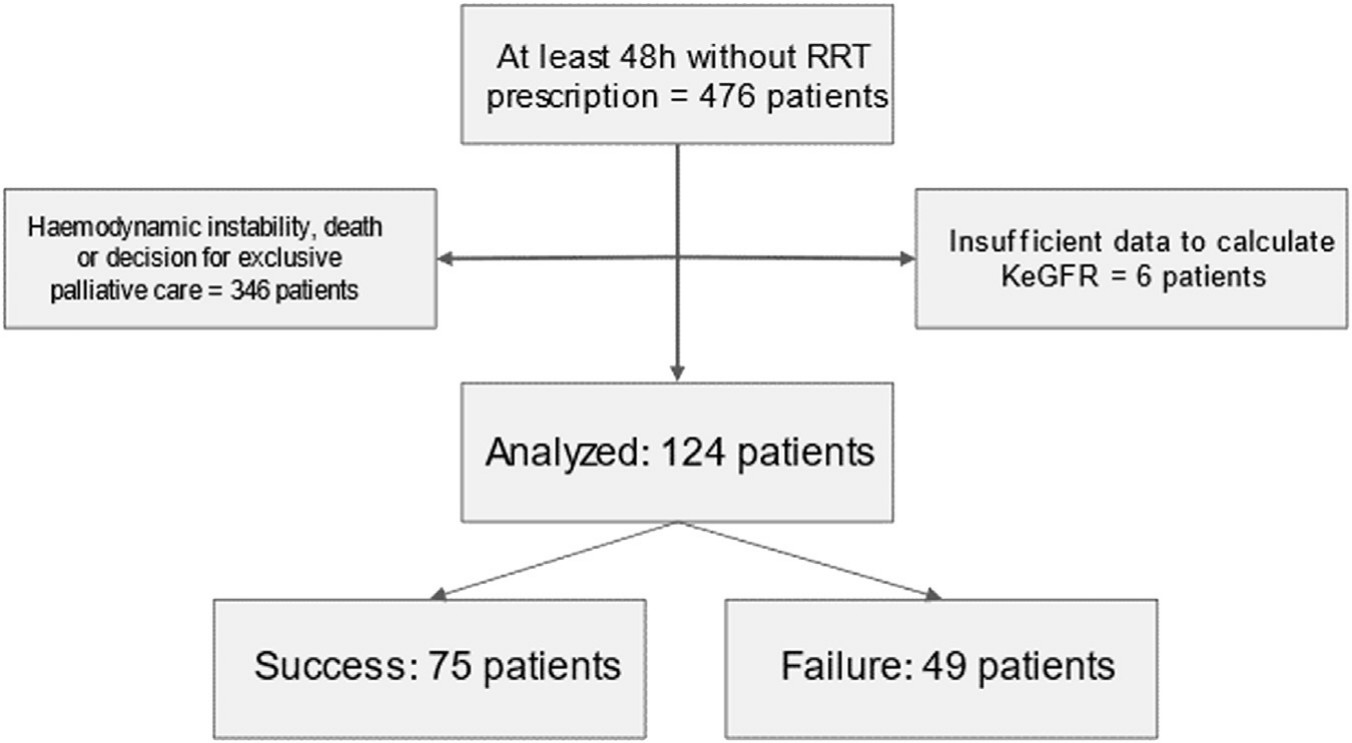
Patient recruitment's flow diagram.

We screened 476 patients. Of those, 346 patients were excluded because RRT was interrupted due to haemodynamic instability, death, or a decision for exclusive palliative care. Extra 6 patients were excluded because of insufficient data to calculate KeGFR. We included 124 patients, 49 in the “Failure” group and 75 in the “Success” group (60.5%) (Fig. 1). Patients in the group “Failure” more commonly presented baseline hypertension, but other comorbidities prevalence were similar between groups. Interestingly, patients who succeeded in RRT discontinuation had both higher SOFA and SAPS severity scores at ICU admission (Table 1). The most common AKI aetiology was COVID19 associated AKI, followed by ischaemia-reperfusion and sepsis (Table 1).

**Table 1:** Baseline patient characteristics, by success in RRT discontinuation.

	[ALL] *n* = 124	Failure *n* = 49	Success *n* = 75	*p*-value	N
Baseline sCr, mg/dL, median [IQR]	0.96 [0.76;1.23]	0.98 [0.69;1.22]	0.96 [0.77;1.23]	0.600	124
Baseline eGFR, mL/min/1.73m^2^ (mean ± SD)	84.5 ± 28.1	85.2 ± 28.2	84.0 ± 28.2	0.826	124
Age, years, median [IQR]	53.5 [44.0;63.0]	57.0 [48.0;65.0]	51.0 [40.0;62.0]	0.135	124
Gender, female, n (%)	41 (33.1%)	14 (28.6%)	27 (36.0%)	0.506	124
Body mass index, Kg/m^2^ median [IQR]	26.0 [23.8;29.2]	26.0 [23.0;29.0]	26.0 [24.0;30.4]	0.470	124
Race, n (%)				0.350	124
White	84 (67.7%)	37 75.5%)	47 (62.7%)		
Black	14 (11.3%)	5 (10.2%)	9 (12.0%)		
Brown	24 (19.4%)	6 (12.2%)	18 (24.0%)		
Other	2 (1.61%)	1 (2.04%)	1 (1.33%)		
Hypertension, n (%)	57 (46.0%)	29 (59.2%)	28 (37.3%)	0.028	124
Diabetes, n (%)	80 (35.5%)	29 (40.8%)	51 (32.0%)	0.417	124
CHF, n (%)	5 (4.03%)	2 (4.08%)	3 (4.00%)	1.000	124
PVD, n (%)	14 (11.3%)	7 (14.3%)	7 (9.33%)	0.574	124
Cirrhosis, n (%)	30 (24.2%)	13 (26.5%)	17 (22.7%)	0.782	124
SAPS, mean ± SD	66.0 ± 14.2	62.7 ± 15.4	68.1 ± 13.0	0.044	124
SOFA, median [IQR]	10.0 [7.00;13.0]	8.00 [5.00;13.0]	11.0 [8.00;13.0]	0.027	124
Main cause of AKI, n (%)				0.928	124
COVID-19	51 (41.1%)	23 (46.9%)	28 (37.3%)		
Sepsis (other than COVID-19)	26 (21.0%)	10 (20.4%)	16 (21.3%)		
SIRS	3 (2.42%)	1 (2.04%)	2 (2.67%)		
Kidney ischaemia	29 (23.4%)	11 (22.4%)	18 (24.0%)		
Presumed AIN	2 (1.61%)	0	2 (2.67%)		
Other	13 (10.5%)	4 (8.2%)	9 (12%)		

AIN, Acute Interstitial Nephritis; BMI, Body Mass Index; CHF, Congestive Heart Failure; COVID-19, Coronavirus Disease; eGFR, Estimated Glomerular Filtration Rate; PVD, Peripheral Vascular Disease; SAPS, Simplified Acute Physiology Score; sCr, Serum Creatinine; SIRS, Systemic Inflammatory Response Syndrome, SOFA, Sequential Organ Failure Assessment Score.

### Interruption day

The occurrence of missing data was low (only 2 variables with missing data above 15%) and we considered all the variables in the database. On the interruption day, half of the patients were using diuretics with no difference between groups. Non-renal SOFA at the time of RRT discontinuation was higher in the “Failure” group when compared with patients in the “Success” group. The ΔnrSOFA was positive in both groups, showing that non-renal SOFA on the day of RRT initiation was higher than on the day of discontinuation, compatible with overall clinical improvement (Table 2). At the time of RRT discontinuation, patients in the group “Success” had higher urine output and more neutral fluid balance (Table 2). In addition, successful patients had lower values of serum urea and potassium and higher levels of blood pH. Values of serum creatinine were not different between groups on the first and second days after RRT interruption, but patients who succeeded had lower levels of sCr on the third day after RRT discontinuation (Table 2).

**Table 2:** Patient characteristics while in RRT and at RRT interruption, by success in RRT discontinuation.

	[ALL] *n* = 124	Failure *n* = 49	Success *n* = 75	*p*-value	N
First RRT modality, n (%)				0.481	124
PIRRT	57 (46.0%)	26 (53.1%)	31 (41.3%)		
CVVHDF	35 (28.2%)	12 (24.5%)	23 (30.7%)		
CVVHD	25 (20.2%)	8 (16.3%)	17 (22.7%)		
CVVH	4 (3.2%)	2 (4.1%)	2 (2.7%)		
Other	3 (2.4%)	1 (2.0%)	2 (2.7%)		
nrSOFA^a^, median [IQR]	5.00 [2.00;7.00]	6.00 [3.00;8.00]	4.00 [1.00;6.00]	0.008	124
ΔnrSOFA^a^, median [IQR]	3.00 [1.00;5.00]	2.00 [0.00;4.00]	4.00 [1.00;6.50]	0.024	124
Diuretic need^b^, n (%)	62 (50.0%)	30 (61.2%)	32 (42.7%)	0.066	124
Urine output^a^, mL/day median [IQR]	1500 [900;2042]	1000 [650;1600]	1700 [1375;2125]	0.001	110
Daily fluid balance^a^, mL/day median [IQR]	332 [-404.25;781]	508 [146;844]	80.5 [-550.50;641]	0.022	110
Serum urea^a^, mg/dL, median [IQR]	118 [83.0;144]	122 [102;148]	109 [71.5;138]	0.013	124
Serum potassium^a^, mEq/L, mean ± SD	4.33 ± 0.67	4.56 ± 0.55	4.18 ± 0.69	0.001	124
Blood pH^a^, median [IQR]	7.40 [7.36;7.44]	7.38 [7.35;7.40]	7.41 [7.38;7.45]	0.003	124
Blood bicarbonate^a^, mmoL/L, median [IQR]	24.0 [21.0;26.0]	23.0 [21.0;25.0]	24.0 [21.1;26.5]	0.228	124
sCr1, mg/dL, median [IQR]	1.94 [1.40;2.71]	1.96 [1.59;2.52]	1.93 [1.36;3.11]	0.904	122
sCr2, mg/dL, median [IQR]	2.72 [1.84;3.51]	2.87 [2.18;3.53]	2.40 [1.62;3.45]	0.096	124
sCr3, mg/dL, median [IQR]	2.70 [1.94;3.67]	3.32 [2.55;4.14]	2.34 [1.71;3.31]	0.001	124
ΔsCr1, mg/dL, mean ± SD	0.64 ± 0.65	0.91 ± 0.67	0.47 ± 0.58	< 0.001	122
ΔsCr2, mg/dL, median [IQR]	0.14 [-0.13;0.43]	0.39 [0.13;0.59]	0.03 [-0.21;0.21]	< 0.001	124
KeGFR1, mL/min, median [IQR]	19.3 [7.62;36.4]	11.2 [3.24;25.2]	25.7 [12.2;44.0]	< 0.001	122
KeGFR2, mL/min, median [IQR]	25.6 [16.0;42.9]	17.2 [12.8;25.9]	33.6 [22.6;49.0]	< 0.001	124
sCr ratio (day 2/day 1), median [IQR]	1.27 [1.11;1.56]	1.44 [1.15;1.72]	1.22 [1.07;1.40]	0.001	122
sCr ratio (day 3/day 1), median [IQR]	1.33 [1.08;1.79]	1.61 [1.30;1.99]	1.19 [0.93;1.57]	< 0.001	122

CVVH, Continuous Venovenous hHaemofiltration; CVVHD, CVVH: Continuous Venovenous Haemodialysis; CVVHDF, Continuous Venovenous Haemodiafiltration; KeGFR1, Kinetic estimated Glomerular Filtration Rate at the second day after RRT discontinuation; KeGFR2, Kinetic estimated Glomerular Filtration rate at the third day after RRT discontinuation; nrSOFA, Non-renal SOFA score; ΔnrSOFA, Variation between nrSOFA on the day of RRT initiation and on the day of RRT discontinuation; PIRRT, Prolonged Intermittent Renal Replacement Therapy; RRT, Renal Replacement Therapy; sCr, Serum Creatinine; sCr1, Serum Creatinine on the first day after at RRT discontinuation; sCr2, Serum Creatinine on the second day after at RRT discontinuation; sCr3, Serum Creatinine on the third day after at RRT discontinuation; ΔsCr1, The difference in sCr levels between the second and the first day after RRT discontinuation, adjusted for 24h; ΔsCr2, The difference in sCr levels between the third and the second day after RRT discontinuation, adjusted for 24h.

Note: To convert serum creatinine in mg/dL to moL/L, multiply by 88.4; urea nitrogen in mg/dL to mmoL/L, multiply by 0.357.

a At the time of RRT discontinuation

b One day before RRT discontinuation.

**Fig. 2:**
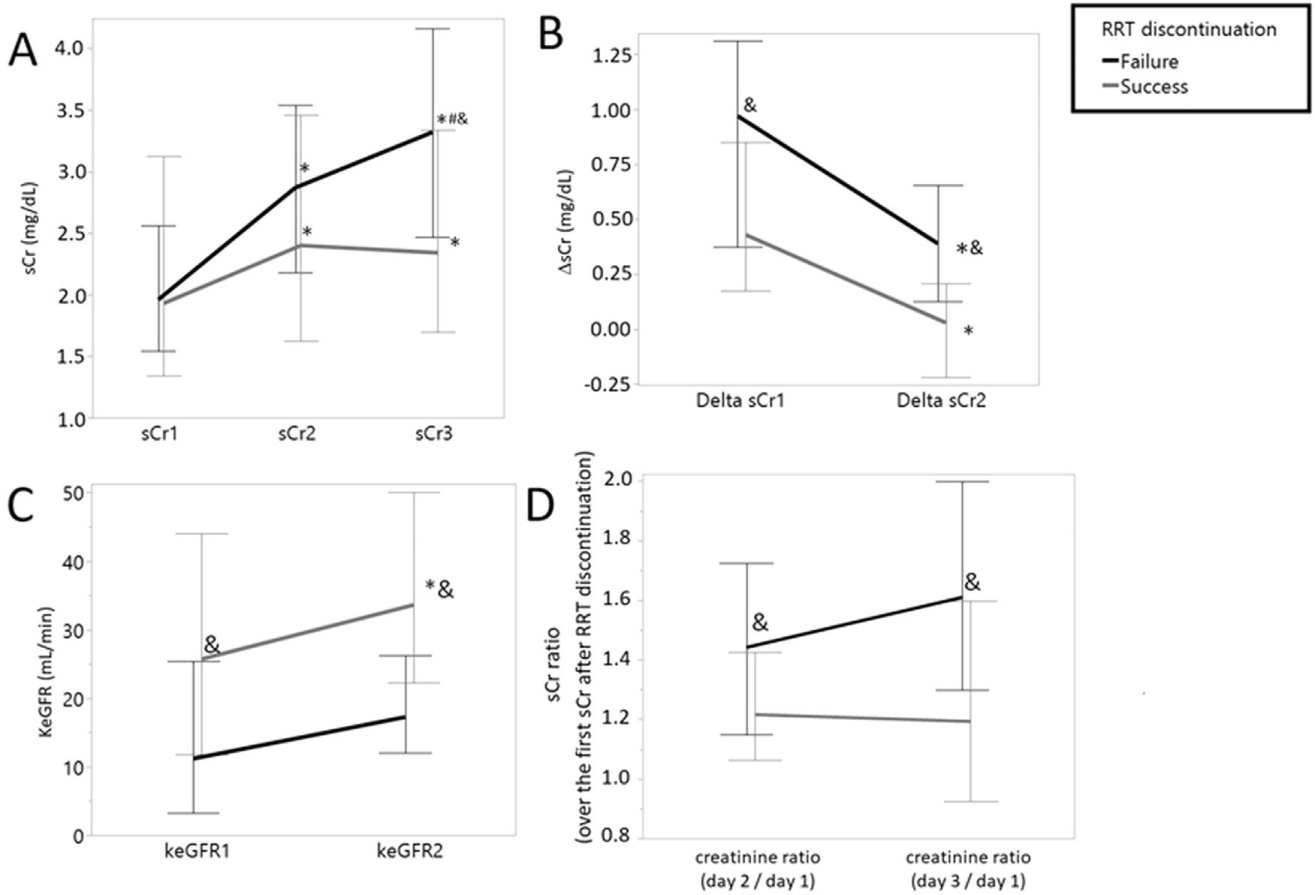
Trajectories of serum creatinine-related variables over the days following RRT discontinuation, by success. Trajectories of serum creatinine-related variables over the days following RRT discontinuation, by success. (A) sCr (mg/dL), (B) ΔsCr (mg/dL), (C) KeGFR (mL/min), (D) sCr ratio (over the first sCr after RRT discontinuation). Values presented as Median [IQR]. *p < 0.05 vs variable on first day, in the same group; # *p* < 0.05 vs variable on second day in the same group; & *p* < 0.05 between groups.

On the first three days after RRT interruption, values of sCr progressively increased in both groups. However, patients in the “Success” group presented a lower increase than patients in the “Failure” group, as demonstrated by ΔsCr1 and ΔsCr2. The KeGFR of both groups were different at the second and third days after RRT discontinuation and both the incremental creatinine ratios (D2/D1 and D3/D1) were different between groups (Table 2). The trajectories of sCr, ΔsCr, KeGFR, and incremental creatinine ratio over the days following RRT discontinuation by success are depicted in Fig. 2.

### Predictors of success

Baseline characteristics and variables collected at the time of RRT discontinuation were tested as potential predictors of RRT weaning success using simple logistic regression models. Results are shown at Table 3. Variables considered predictors of the outcome in the univariable models were previous hypertension, SAPS, nrSOFA, ΔnrSOFA, diuretic need, urine output, daily fluid balance, serum urea, serum potassium, blood pH, sCr3, ΔsCr1, ΔsCr2, KeGFR1, KeGFR2, sCr ratio day 2/day 1 and sCr ratio day 3/day 1. Factors predicting Success were the absence of baseline hypertension, high SAPS score, low nrSOFA at the time of discontinuation, high ΔnrSOFA (showing higher changes between nrSOFA on the day of RRT initiation and on the day of RRT discontinuation), no need of diuretic at the time of RRT discontinuation, high urine output, low values of fluid balance, low urea and potassium levels, high pH, low value of sCr 3 days after RRT discontinuation, low values of ΔsCr (showing less increase in sCr within two subsequent days), high values of KeGFR and low incremental sCr ratio (Table 3).

**Table 3:** Univariable logistic regression models to predict success in RRT discontinuation.

Risk factor	OR (95% CI)	*p*-value	c-statistic
Baseline sCr	1.29 (0.56; 3.17)	0.56	0.53
Baseline eGFR	0.998 (0.99; 1.01)	0.82	0.51
Age	0.98 (0.95; 1.005)	0.13	0.58
Gender	0.71 (0.32; 1.53)	0.39	0.54
BMI	1.01 (0.94; 1.08)	0.86	0.54
Race	Black: 1.42 (0.44; 4.59)	0.37	0.57
	Brown: 2.36 (0.85; 6.55)		
	Other: 0.79 (0.05; 13.01)		
Hypertension	0.41 (0.19; 0.85)	0.02	0.61
Diabetes	0.68 (0.321; 1.45)	0.32	0.54
CHF	0.98 (0.16; 7.64)	0.98	0.50
PVD	0.62 (0.20; 1.88)	0.40	0.52
Cirrhosis	0.81 (0.35; 1.87)	0.62	0.52
SAPS	1.03 (1.002; 1.06)	0.04	0.60
Main cause of AKI	Meaningless	0.99	0.57
First RRT modality	CVVHDF: 1.61 (0.68; 3.92)	0.99	0.56
	CVVHD: 1.78 (0.68; 4.98)		
	CVVH: 0.84 (0.09; 7.39)		
nrSOFA^a^	0.86 (0.77; 0.97)	0.01	0.64
Δ nrSOFA^a^	1.13 (1.03; 1.26)	0.01	0.62
Diuretic need^b^	0.47 (0.22; 0.97)	0.044	0.59
Urine output^a^	1.0007 (1.000; 1.001)	0.005	0.69
Daily fluid balance^a^	0.9995 (0.9990; 0.9999)	0.04	0.63
Serum urea^a^	0.99 (0.98; 0.999)	0.04	0.63
Serum potassium^a^	0.39 (0.20; 0.70)	0.002	0.68
Blood pH^a^	2094 (9.04; 1079089)	0.01	0.66
Blood bicarbonate^a^	1.06 (0.96; 1.18)	0.23	0.56
sCr1	1.13 (0.84; 1.57)	0.41	0.51
sCr2	0.94 (0.76; 1.18)	0.61	0.59
sCr3	0.79 (0.62; 0.99)	0.05	0.68
ΔsCr1	0.31 (0.15; 0.58)	0.0005	0.69
ΔsCr2	0.10 (0.03; 0.25)	0.00002	0.77
KeGFR1	1.03 (1.01; 1.06)	0.002	0.69
KeGFR2	1.05 (1.03; 1.09)	0.0001	0.76
sCr ratio (day 2/day 1)	0.15 (0.04; 0.43)	0.001	0.67
sCr ratio (day 3/day 1)	0.16 (0.06; 0.38)	0.00006	0.73

BMI, Body Mass Index; CHF, Congestive Heart Failure; eGFR, Estimated Glomerular Filtration Rate; KeGFR1, Kinetic Estimated Glomerular Filtration rate at the second day after RRT discontinuation; KeGFR2, Kinetic Estimated Glomerular Filtration rate at the third day after RRT discontinuation; nrSOFA, non-renal SOFA score; ΔnrSOFA, Variation between nrSOFA on the day of RRT initiation and on the day of RRT discontinuation; PVD, Peripheral Vascular Disease; SAPS, Simplified Acute Physiology Score; RRT, Renal Replacement Therapy; sCr, Serum Creatinine; sCr1, Serum Creatinine on the first day after at RRT discontinuation; sCr2, Serum Creatinine on the second day after at RRT discontinuation; sCr3, Serum Creatinine on the third day after at RRT discontinuation; ΔsCr1, The difference in sCr levels between the second and the first day after RRT discontinuation, adjusted for 24h; ΔsCr2, The difference in sCr levels between the third and the second day after RRT discontinuation, adjusted for 24h.

a At the time of RRT discontinuation

b One day before RRT discontinuation.

**Fig. 3:**
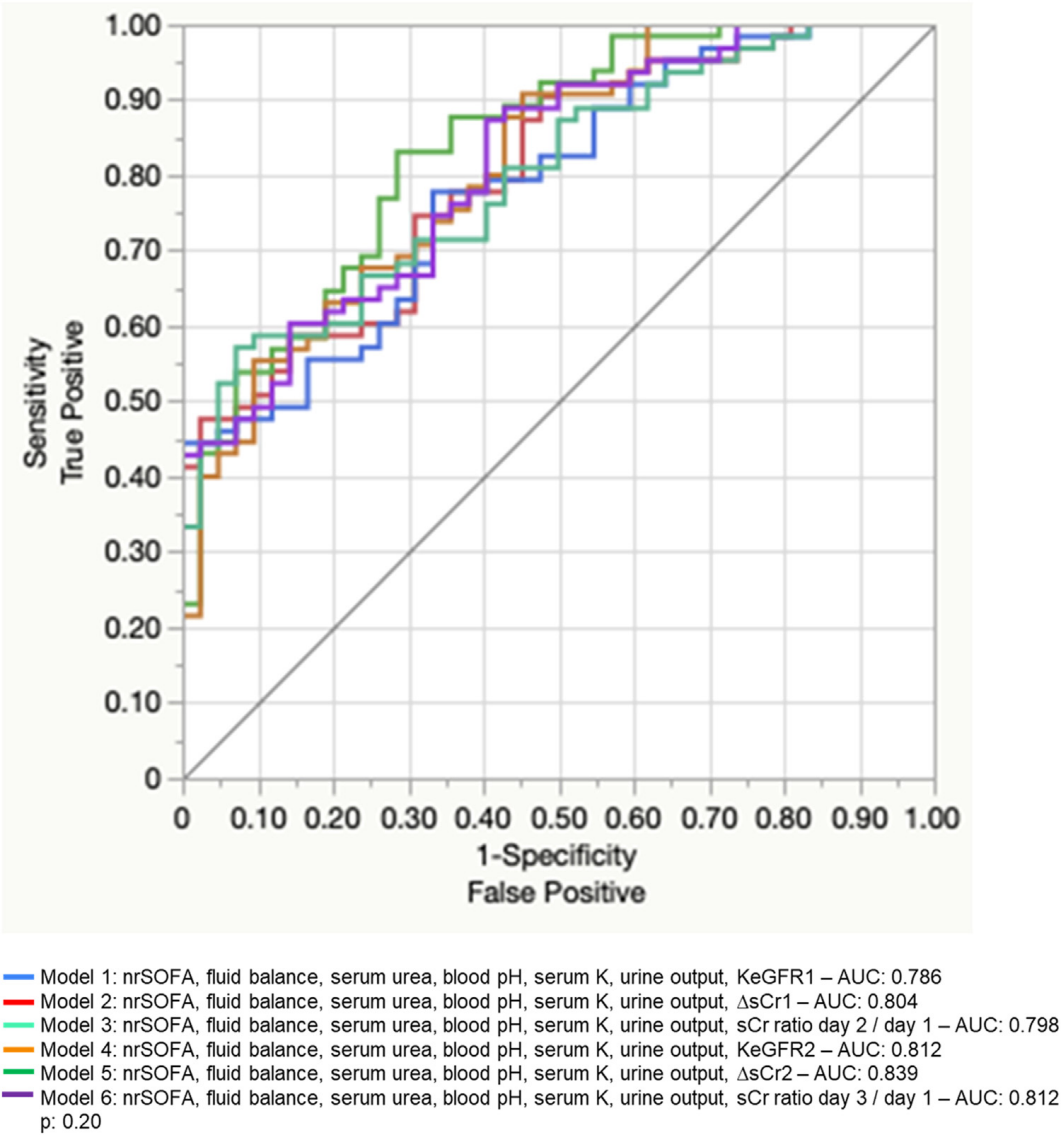
Multivariable model comparison to predict success in RRT discontinuation.

To generate multivariate models, we selected one of the clinical score variables (among SAPS, nrSOFA, Δ nrSOFA), urine output, daily fluid balance, serum urea, serum potassium, blood pH, and one variable related to sCr (among sCr3, ΔsCr1, ΔsCr2, KeGFR1, KeGFR2, creatinine ratio [day 2/day 1] and creatinine ratio [day 3/day 1)]). We chose nrSOFA among clinical score variables because it was the easiest to calculate and correlated very well with the outcome. The variable need of diuretics was not included because it is clinically related to urine output, which is a more relevant parameter to be analysed. We decided not to include hypertension in the model because this information is usually difficult to timely obtain in a reliable manner in critically ill patients. As we wanted to compare the KeGFR with the simple variation in serum creatinine between two consecutive days and the incremental sCr ratio at the time of RRT interruption to predict successful weaning, we generated six multivariable logistic regression models, each one including one of the creatinine-related predictors (comprising ΔsCr1, ΔsCr2, KeGFR1, KeGFR2, creatinine ratio [day 2/day 1] and creatinine ratio [day 3/day 1]). We decided not to include single values of sCr because this variable was already included in the results of ΔsCr, KeGFR, and sCr ratio. Multivariable models to predict success in discontinuation were compared using ROC curves and the result is depicted in Fig. 3 and Table 4. Models were similar in prediction performance.

**Table 4:** Multivariable logistic regression models to predict Success in RRT discontinuation.

Risk factor	Model 1 – c-statistic 0.786		Model 2 – c-statistic 0.804		Model 3 – c-statistic 0.798		Model 4 – c-statistic 0.812	
	OR (95% CI)	*p*-value	OR (95% CI)	*p*-value	OR (95% CI)	*p*-value	OR (95% CI)	*p*-value
nrSOFA^a^	**0.85 (0.73; 0.99)**	**0.04**	0.87 (0.74; 1.01)	0.06	0.88 (0.75; 1.03)	0.11	**0.83 (0.71; 0.98)**	**0.02**
UO^a^	1.00 (1.00; 1.00)	0.12	1.00 (1.00; 1.00)	0.08	1.00 (1.00; 1.00)	0.09	1.00 (1.00; 1.00)	0.23
Daily FB^a^	1.00 (1.00; 1.00)	0.36	1.00 (1.00; 1.00)	0.39	1.00 (1.00; 1.00)	0.50	1.00 (1.00; 1.00)	0.25
Serum U^a^	1.00 (0.99; 1.01)	0.76	1.00 (0.98; 1.01)	0.61	0.99 (0.98; 1.00)	0.26	1.00 (0.99; 1.01)	0.69
Serum K^a^	**0.39 (0.16; 0.96)**	**0.04**	**0.40 (0.16; 0.96)**	**0.03**	0.42 (0.17; 1.02)	0.05	**0.40 (0.16; 0.99)**	**0.04**
Blood pH^a^	57.67 (0.04; 86826.6)	0.28	84.24 (0.05; 133270.3)	0.22	152.98 (0.09; 269551.6)	0.17	32.87 (0.02; 67337.7)	0.36
KeGFR1	**1.03 (1.00; 1.05)**	**0.04**						
ΔsCr1			**0.36 (0.15; 0.85)**	**0.01**				
sCr ratio (day 2/day 1)					**0.20 (0.05; 0.85)**	**0.02**		
KeGFR2							**1.04 (1.01; 1.08)**	**< 0.01**
ΔsCr2								
sCr ratio (day 3/day 1)								
**Risk factor**	**Model 5 – c-statistic 0.839**		**Model 6 – c-statistic 0.812**					
	**OR (95% CI)**	***p*-value**	**OR (95% CI)**	***p*-value**				

nrSOFA^a^	**0.84 (0.71; 1.00)**	**0.04**	0.89 (0.75; 1.04)	0.14				
UO^a^	1.00 (1.00; 1.00)	0.15	1.00 (1.00; 1.00)	0.13				
Daily FB^a^	1.00 (1.00; 1.00)	0.50	1.00 (1.00; 1.00)	0.54				
Serum U^a^	0.99 (0.98; 1.00)	0.07	0.99 (0.98; 1.00)	0.15				
Serum K^a^	0.49 (0.20; 1.20)	0.11	**0.40 (0.16; 0.99)**	**0.04**				
Blood pH^a^	188.74 (0.05; 685805.7)	0.20	263.54 (0.10; 672343.4)	0.14				
KeGFR1								
ΔsCr1								
sCr ratio (day 2/day 1)								
KeGFR 2								
ΔsCr2	**0.08 (0.02; 0.35)**	**< 0.01**						
sCr ratio (day 3/day 1)			**0.16 (0.05; 0.55)**	**< 0.01**				

FB, Fluid Balance; K, Potassium; KeGFR1, Kinetic Estimated Glomerular Filtration Rate at the second day after RRT discontinuation; KeGFR2, Kinetic Estimated Glomerular Filtration rate at the third day after RRT discontinuation; nrSOFA, Non-renal SOFA score; sCr, Serum Creatinine; ΔsCr1, The difference in sCr levels between the second and the first day after RRT discontinuation, adjusted for 24h; ΔsCr2, The difference in sCr levels between the third and the second day after RRT discontinuation, adjusted for 24h; U, Urea; UO, Urine output.

a At the time of RRT discontinuation.

### Model development and validation

**Fig. 4:**
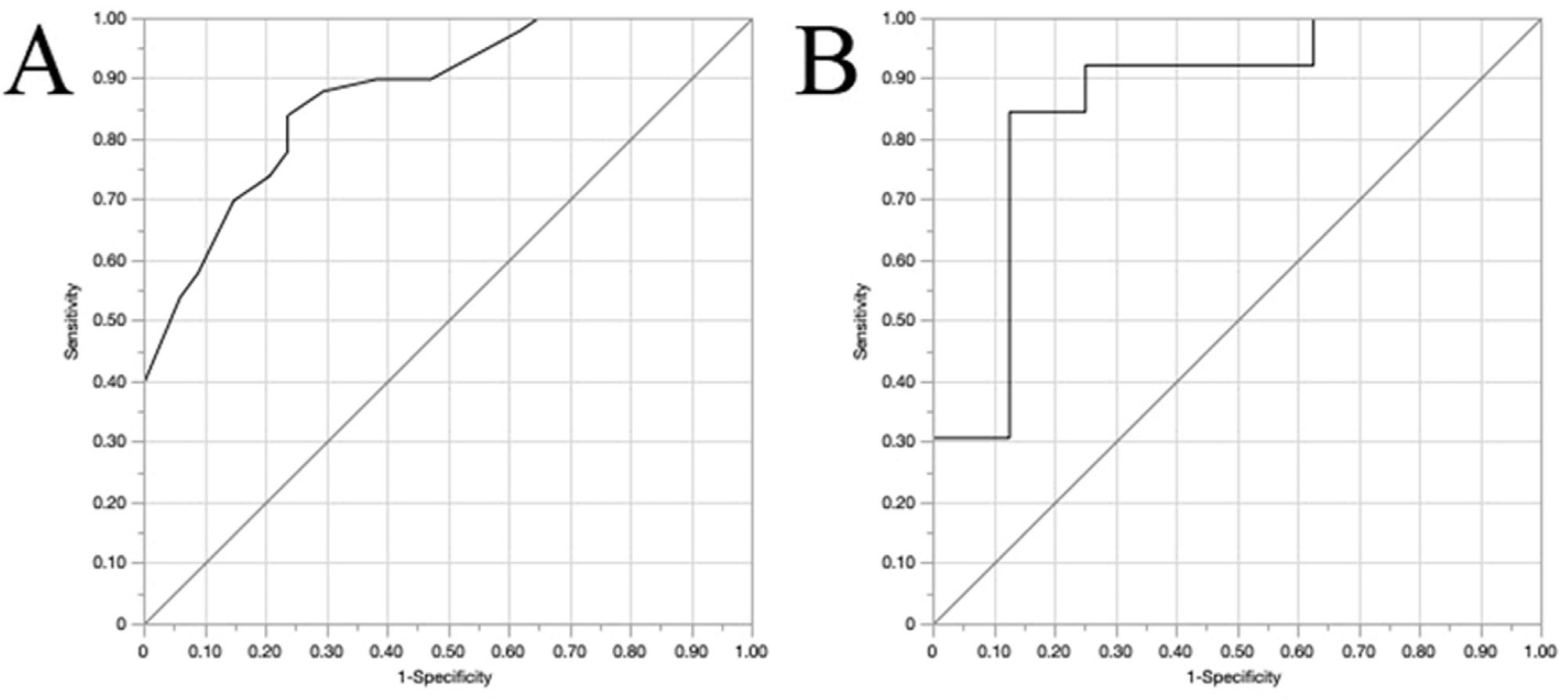
Training and internal validation of the proposed clinical score to predict success in RRT discontinuation. (A) Training curve ‒ AUC 0.87 (95% CI 0.74‒0.92); (B) Validation curve ‒ AUC 0.86 (95%CI 0.76‒1.00).

As there was no difference among models, we chose a final model that comprised variables collected earlier, known after only two days after RRT discontinuation (Models 1, 2 and 3). Model 2 was chosen because of an AUC under ROC curve slightly better than Models 1 and 3. Variables used to generate Model 2 were transformed into binomial variables according to the optimal cutoff originated by the ROC curves at each univariable model. Based on this, we generated a new multivariable model using those binomial variables as predictors of “Success” (Table 5). The Odds Ratio (OR) obtained for each variable was rounded to the nearest integer and we determined the value of each variable to generate a clinical score to predict success in RRT discontinuation (Table 5). Internal validation was performed to test the ability of the clinical score to predict success in RRT discontinuation. The training and validation models were obtained by binomial generalised regression model with Lasso estimation and 5-fold cross validation method (Fig. 4). The optimal cutoff to predict success using the score generated would be 11 points (Youden index). At the training model, sensitivity, specificity, ´Positive Predictive Value (PPV), and Negative Predictive Value (NPV) were 84%, 76.5%, 84% and 76.5%. At the internal 5-fold validation model, sensitivity, specificity, Positive Predictive Value (PPV) and Negative Predictive Value (NPV) were 92.3%, 75%, 85.7% and 85.7%. The likelihood of RRT weaning success was generated and is shown at Table 6.

**Table 5:** Multivariable model using binomial predictors to generate success in RRT discontinuation score. Variables were selected based on Model 2 and values were based on ROC curves generated after univariable regression models.

c-statistic: 0.88	OR (95% CI)	*p*-value	score
Urine output > 1350 mL/day	4.50 (1.46; 13.90)	0.01	4
nrSOFA ≤ 4	3.56 (1.18; 10.75)	0.02	4
Fluid balance ≤ 86 mL/day	2.93 (0.93; 9.24)	0.07	3
Serum urea ≤ 91 mg/dL	7.58 (1.69; 33.93)	0.01	8
pH > 7.40	1.81 (0.58; 5.63)	0.30	2
Serum K ≤ 4.3	4.84 (1.53; 15.27)	0.01	5
ΔsCr1 ≤ 0.87mg/dL	2.71 (0.85; 8.66)	0.09	3
Final complete score			29

K, Potassium; nrSOFA, Non-renal SOFA score; ΔsCr1, The difference in sCr levels between the second and the first day after RRT discontinuation, adjusted for 24h.

Note: To convert serum creatinine in mg/dL to moL/L, multiply by 88.4; urea nitrogen in mg/dL to mmoL/L, multiply by 0.357.

**Table 6:** Likelihood of RRT discontinuation success based on the score obtained in the proposed model.

Score	Likelihood of success
0	4.9%
5	18.1%
10	48.4%
15	80.0%
18	90.5%
20	94.4%
25	98.6%

## Discussion

In this study, we found that the success in RRT independence at 7 days after AKI-associated RRT discontinuation can be predicted by the KeGFR calculated using sCr levels on the first and second days (KeGFR1) as well as on the second and third days after RRT discontinuation (KeGFR2). The incremental sCr ratio day 2/day 1 and day 3/day 1 had similar performance and the simple variation in serum creatinine between 2 consecutive days after RRT discontinuation could also similarly predict the outcome, either when the difference between sCr levels on the first and second days (ΔsCr1) or the difference between sCr levels on the second and third days was considered (ΔsCr2).

We demonstrated that there is no need for a decrease in sCr to predict recovery and successful discontinuation. In fact, even for successful patients the raw value of sCr continued to increase between the first and second days after RRT interruption (although the rate of increase was lower in successful patients). On day 3, sCr values started to reach a steady pattern and were similar to values at day 2 in patients who succeeded, but not in patients who failed.

Both KeGFR and incremental creatinine values have already been shown to predict RRT discontinuation in prior studies,^13^^,^^15^ but they have never been compared before. In this study, we showed that any strategy may have equivalent performances to determine if a patient will be able to keep RRT-independence one week after initial RRT discontinuation.

No previous study showed an acceptable success RRT discontinuation rate with urine output lower than 400 mL daily. Thus, it will be very unlikely that any patient would be discontinued from RRT due to the expectation of renal recovery with a urine output lower than that. The Standardized Clinical Assessment and Management Plans (SCAMP) Study discontinuation algorithm recommends a urine output cut-off of at least 500 mL per day.^18^ In this study we evaluated urine output as a predictor of success and the optimal threshold found here was 1350 mL daily, which could be explained by the critical illness scenario.

Previous studies assessing recovery from AKI and RRT discontinuation have not studied non-renal SOFA score on the day of discontinuation. In this study, we showed that this variable was importantly associated with success (both as the raw nrSOFA value at the time of RRT interruption and the change in SOFA score over the days represented by the variable ΔnrSOFA). The nrSOFA can translate the bedside clinical impression into an objective measurable data and contribute to guide RRT discontinuation.

In multivariable models including non-renal SOFA score, daily fluid balance, urea and potassium levels, blood pH and daily urine output, all the six mentioned sCr-related variables had similar performance in prediction of RRT discontinuation success. Models constructed using later sCr values, such as KeGFR2, ΔsCr2 and sCr ratio (day 3/day 1), seemed to be slightly better than models using earlier sCr levels. However, the difference among them was meaningless and we gave preference to models that would provide earlier clinical information to guide medical decisions and prevent unnecessary maintenance of RRT. The second step to guide model choice was model performance and we showed that the one including ΔsCr1 (Model 2) presented a slightly higher area under ROC than the models including KeGFR1 (Model 1) or sCr ratio (day 2/day 1) (Model 3). The difference was minimal and we could select any of those, although ΔsCr1 and sCr ratio (day 2/day 1) are easier to apply without an online calculation tool, compared to KeGFR1, thus the choice for a model including a simple variation in daily sCr would facilitate clinical use. While ΔsCr provides the direction of change in GFR but not the degree of GFR, the KeGFR provides the magnitude of GFR and not the direction of change. To see the direction of change using KeGFR, three values of sCr would be needed, to calculate two subsequent KeGFR levels. Christiadi et al demonstrated that the ratio of KeGFR at any time to baseline KeGFR could timely predict AKI.^19^ A decrease of the ratio greater than 10% had a positive predictive value of 63% and a negative predictive value of 80% in future clinical diagnosis using sCr and urine output KDIGO criteria.^1^^,^^19^ In our study, both direction (as demonstrated by ΔsCr) and magnitude (as demonstrated by KeGFR) of change in GFR were interchangeable in RRT discontinuation success prediction.

The ΔsCr may not replace the KeGFR in all clinical settings. The knowledge about the amount of kidney function may be important to adjust medication dosing in patients with AKI,^20^ for example. However, the role of KeGFR in clinical practice has not been completely established yet. KeGFR was not equivalent to measured creatinine clearance in previous studies evaluating antibiotics dosage in critically ill patients^21^ and different KeGFR equations, including the one used here and proposed by Chen,^14^ were compared to iohexol clearance among patients with shock, none of them being accurate.^22^ Maybe better KeGFR equations to precisely estimate GFR in AKI and AKI recovery phase are still necessary.

In this study, we suggested a clinical score to predict success in RRT discontinuation. The model generated using the clinical score as a predictor had a good internal validation provided by the 5-fold validation statistical tool. However, external validation could not be provided here.

The KDIGO guidelines recommend RRT interruption when intrinsic kidney function has recovered to the point that it is adequate to meet patient needs.^1^ However, different patients have different needs. While stable patients could dismiss haemodialysis uneventfully with a given glomerular filtration rate, other patients in hypercatabolic states could develop refractory hyperkalemia with the same level of GFR. Patients with heart diseases might not tolerate RRT discontinuation regardless of GFR if urine output is not enough to prevent positive fluid balance. These situations would never be correctly evaluated with a single variable approach, and this is the strength of a clinical score to indicate possible patient needs.

Our model included widely available predictors that are clinically relevant to discontinuation success, such as urine output, serum potassium, serum urea and creatinine rise ratio, and combines them into a simple clinical score to facilitate decision making. To the best of our knowledge, this study provides the first clinical score to predict success on haemodialysis discontinuation in critically ill patients with AKI. It presents a promising clinical tool that may help doctors to make better decisions regarding AKI management.

In the studied population, 90% of patients with ≥18 points (out of 29 points) in the clinical score proposed were successfully withdrawn from RRT 7 days after RRT interruption. This finding might be a striking contribution if further confirmed in future studies. Many patients who interrupt AKI-related RRT have to resume therapy in the following 2‒3 days. A clinical score that sorts out who these patients are would be very informative. Patients with high score levels, e.g., ≥ 18 points, maybe would be able to have their vascular access removed. In settings with low RRT machine availability, RRT machines that would be required for those patients would possibly be reallocated, allowing better usage of limited resources.

Although there are many studies regarding RRT initiation on AKI,^4^^,^^23^, ^24^, ^25^, ^26^ there are few, small and mostly retrospective studies about RRT interruption. Many studies investigated variables that are still not routinely available in ICU, such as daily urinary urea excretion[10] and urine NGAL,^9^ while our study included only clinical variables that are easily assessed at any ICU, regardless of resources availability.

Our study has several limitations. Firstly, it is a retrospective observational single-centre study, so results might not be generalised to some populations. Secondly, despite our sample size being larger than most RRT discontinuation studies, it is still not a large cohort. This can lead to biases caused by, for example, the high prevalence of COVID-19 patients in our cohort, which does not correspond to the current reality of most ICUs after widespread vaccination. Finally, our score was validated internally with the 5-fold validation method but still lacks a prospective external validation cohort before it can be implemented in clinical practice and proposed as a clinical utility tool.

New biomarkers such as urine NGAL are not readily available in our centre and were not included in our analysis. It is important to point out that new biomarkers in the setting of RRT discontinuation are being studied. In 2021, Daniels et al.^27^ published a study that identified more than 20 serum biomarkers that were independently associated with success in RRT discontinuation. These findings suggest that future scores should include biomarkers as soon as their effectiveness is proven, and they become widely available.

## Conclusion

Both KeGFR and simple variation in serum creatinine between 2 consecutive days after RRT discontinuation might predict success in RRT discontinuation. The suggested clinical score developed based on these variables might be a useful clinical decision tool to guide medical decisions, but still requires validation in larger studies before it can be implemented in clinical practice.

## Conflicts of interest

The authors declare no conflicts of interest.
